# 

**Published:** 1901-07

**Authors:** 


### BOOKS RECEIVED.

Etidorpha or the End of Earth. The Strange History of a Mysterious Being and the Account of a Remarkable Journey. By John Uri Lloyd, author of Stringtown on the Pike. With many illustrations by J. Augustus Knapp. Eleventh edition revised and enlarged. New York: Dodd, Mead & Co. 1901. [Price, $1.50.]

Transactions of the American Associations of Obstetricians and Gynecologists. Thirteenth annual meeting held at Louisville, September 18, 19 and 20, 1900. William Warren Potter, M. D., Secretary. Philadelphia: William J. Dornan, Printer. 1901.

Progressive Medicine, Vol. II., June, 1901. A Quarterly Digest of Advances, Discoveries and Improvemeuts in the Medical and Surgical Sciences. Edited by

Hobart Amory Hare, M. D., Professor of Therapeutics and Materia Medica in Jefferson Medical College of Philadelphia. Octavo, handsomely bound in cloth, 460 pages, with 81 engravings and one full-page plate. Issued Quarterly, Philadelphia and New York: Lea Brothers & Co. 1901. [Price, $10.00 per year.]

A System of Physiologic Therapeutics. A Practical Exposition of the Methods, Other than Drug-Giving, Useful in the Treatment of the Sick. Edited by Solomon Solis Cohen, A. M., M. D., Professor of Medicine and Therapeutics in the Philadelphia Polyclinic; Lecturer on Clinical Medicine at Jefferson Medical College, etc. Volume II. Electrotherapy, by George W. Jacoby, M. D., Consulting Neurologist to the German Hospital, New York City; to the Infirmary for Women and Children, etc. In two books: Book II. Diagnosis; Therapeutics. Illustrated. Philadelphia: P. Blakiston's Son & Co. 1901. [Price, eleven volumes, $22.00 net.]

Operative Surgery. By Joseph D. Bryant, M. D., Professor of the Principles and Practice of Surgery, Operative and Clinical Surgery, University and Bellevue Hospital Medical College; Visiting Surgeon to Bellevue and St. Vincent's Hospital, etc. Vol. II. Operations on Mouth, Nose and Esophagus, the Viscera connected with the Peritoneum, the Thorax and Neck, Scrotum and Penis, and miscellaneous operations. 8vo, pp. 729. 857 illustrations, including 40 colored ones. New York: D. Appleton & Co. 1901.

Transactions of the Twenty-second Annual Meeting of the American Laryngological Association held in the City of Washington, D C., May 1, 2 and 3, 1900. Dr. James E. Newcomb, Secretary. New York: Carey Printing Co. 1901.

---

### LITERARY NOTES.

### Index to Volume XL.

(New Series.)

PAGE.

ABBOTT, FRANK W., In Memoriam . . 761
Abdominal Diseases, Surgical Diagnosis of Right Side 573
Abdominal Section, Post-Operative Treatment of . 36
Section, Wearing Belts After . 846
Abortion Cases, Dying Declaration in . . . . . . . . . . . 117
Acidity, Powder for Gastric . . . . 918
Adolescence, Medical Treatment During . . . . . 845
Adrenalin, Clinical Experience with . 918
Albany Hospital, Photographing Operations at . . . . . . . . 527
Albuminuria, Renal Casts Without . 188
Alcoholism—A Crime or a Disease? 493
Amenorrhea, For . . 920
Treated with Ergoapiol, 443
Anesthesia by Nitrous Oxide Gas and Ether . . . . 87
Anesthetic, New Local for Ear . . 192
Aneurism, Cirsoid, of the Ear 397
Anginas, Membranous, Caused by Microörganisms Other than the Klebs-Löffler Bacillus . 312
Animal Nature, Application of Grosser in Medicine . . . 864, 743
Antitoxin in Cerebrospinal Meningitis . . . . . . 177
Appendicitis, Early and Late Operations for . 23
Increase of, Among Women, Joseph Price's Explanation, 925
Army Bill Amended . . . . 450
Asthma, Pathology of . 815
Autoinfection in Diseases of Children, 305

BACTERIOLOGICAL laboratory . . 215
Bainbridge, W. S., Fracture and Dislocation of Inferior Maxilla . . . . . . . . . . 166

PAGE.

Ballou, E. H., Country Health Officer . . 488
Bathhouse in Buffalo, Second Free . 526
Bayliss, A. W., How are we Educating our Children? . . . . . 888
Beahan, A. L., Abdominal Drainage, 330
Right Side Abdominal Diseases 573
Benedict, A. L., Analysis of Proteids in Stomach Contents . . . . 669
Bennett, M. L., Empyema in Young Children . . . 567
Bissell, William G., Formaldehyde Gas . . 495
Membranous Anginas Caused by Microörganisms . . . 312
Bladder, Removal of, for Malignant Disease . . . . . . . . . . . 900
Blood Examinations . . . 319
Specific Gravity of, and Percentage of Hemoglobin . . 153
Boils, To Abort . . . . . 275
Books Received 72, 151, 226, 301, 378, 466, 546, 624, 710, 789, 870, 942
Bottini's Operation . . . 191
Bronchitis, For Chronic . . . . 690
Burns, Topical Application for . . . 193
Busch, Kerr and Filsinger, Relation of Specific Gravity of Blood to Percentage of Hemoglobin . . . 153

CALCULUS, Prostatic . . . . 189
Cancer, Buffalo Laboratory Investigation of . . . . 549
Cancerous Process, Nature of . 873
Carcinoma of the Uterus . 182
Carstens, J. H., Value of Dry Sterilised Catgut . . . . . . 261
Cataract, Absorption of Senile . . . 109
Catgut, Value of Dry Sterilised . 261
Chase, Walter B., Post-Operative Treatment of Abdominal Section . 36
Cheesman, William S., Cirsoid Aneurism of the Ear . . . . . 397

PAGE.

Children, How are we Educating? . 888
Cholelithiasis from the Surgical Viewpoint . . . . . 385
Christian Science and Knights of Honor . . . 59
Institute, Charter Denied . . . 853
Church, W. F., Abuse of Quinine . 98
Clark, L. Pierce, Bromides in Epilepsy . . . . . . . 469
Clinton, Marshall, Fractures of the Nose . . . . . . . . 811
Code Worship, Commentary on . 214

**College and Hospital Notes:**

Albany Hospital . . . . 67, 861, 9 ‘3
Albany Medical College Alumni Association . . . . . 140
Batavia Hospital . . . . . . . 705
Brigham Hall . . . . 612
Buffalo Charity Eye, Ear and Throat Hospital . . . 612
Buffalo Eye and Ear Infirmary 535
Buffalo General Hospital . . . . . . . . 362, 779, 780
Buffalo Homeopathic Hospital . 862
Buffalo Hospital Sisters of Charity . . . . 533
Buffalo State Hospital . . . 535
Cancer Hospital . . . . . 457
College of Medicine, Syracuse University . 776
College of Physicians and Surgeons, Chicago . . . 363
Consumption Hospital . 457, 704
Cornell University Medical College . . . . 935
Dental Department, U. of B. . . . . . . . . . 286, 861
Emergency Hospital at Pan-American Exposition . . . 701
Fitch Hospital . . 705
German Hospital Dispensary 779
Gowanda State Hospital . . 935
Kentucky School of Medicine . 66
Laboratory of Pathology, U.of B. 612
Manhattan Maternity Hospital . 705
Medical College Western Reserve University . . . . 534
Mercy Hospital, Pittsburg . 705
New Emergency Hospital . 363, 455
New German Hospital . 456, 704
New York School of Clinical Medicine . . . . . 533
New York Skin and Cancer Hospital . . . . . 363, 706
New York State Hospital for Crippled and Deformed Children . . . . . . . . . . . 780

PAGE.

New Orleans Polyclinic 780
Niagara Falls Memorial Hospital . . . . . . . 861
Northwestern University Medical School . . . . 220
Oak Grove Hospital, Flint, Mich . . . . . . . 534
Sheppard and Enoch Pratt Hospital . . 534
State Hospital for Tuberculosis, 861
University of Buffalo . . . . 140, 220, 286, 934
University of Pennsylvania . . 612
University of Pennsylvania Alumni Association . . . 456

Communications, Privileged . . . . 58
Conception, Prevention of . 117
Congdon, Charles E., Obstetrics from a Gynecological Standpoint 260
Toilet of the Peritoneum, 405
Conjunctivitis, Simple . . . . . 194
Constipation in Children . . . . . 196
Consumptives, Census of . . . . 924
Isolation of . . . . 59

**Correspondence:**

A Correction—Geo. T. Stevens, 690
Ethnology and Archeology at the Fair—A. L. Benedict . . 49
Inherited Tendency to Appendicitis—W. H. DeWitt 925
Medical Mirror Prize Contest—Medical Mirror . 277
New York School of Clinical Medicine—Marcus Kenyon . 49

Cough, For a Tickling . . . . 277
Coughs, Their Suppression and Cure, 685
Crockett, M. A., Toxemia of Pregnancy . . . . . . . . . . . . 736
Croup . . . . . . 195
Cutaneous Growths, For Small . . . 275
Cystitis, Acute . . . . . . . . . . 195

DAGGETT, BYRON H., Physiology of the Kidney . . . . 898
Death and Insuranca . . . 59
Sentence and Insurance, 59
Dermatitis Exfoliativa . . . . . 481
Diarrhea in Children . . . . 274
Treatment of . . 444
Dickinson, William L., Excision of Hemorrhoids . 651
Digestion, Functional Disorders of . 653
Dormiol, The Administration of . . 919

PAGE.
Dowd, J. Henry, Gonorrheal Rheumatism . . . . . . . . . 92
Impressions Regarding Genito-urinary Diseases, 563
Drainage, Abdominal . . . . 330
Dysmenorrhea . . . . . . . . . 197

ECTOPIC GESTATION, Symptomatology of Early . . . . 30
Empyema in Young Children . 567
Enuresis . . . . . 275
Epilepsy, Bromides in . . . . . 469
Santonin in . . . . . . 277
Eye, Dionin in Diseases of . . . . 110

FIBROID, Removal of Pedunculated . . . . . . . 412
Foods, Artificial . . . . 183
Formaldehyde . . . . . . . 553
Gas . . . . . . 495
Fronczak, F. E., Alcoholism, a Crime or a Disease? . 493
Medical Glimpses of Lands Across the Seas . . . 255
Frye, Maud J., Symptomatology of Early Ectopic Gestation . . . 30

GALVESTON HURRICANE 357
Picturesque . 282
Gastric Catarrh, Blood Cure of 267
Gastritis, Acute . . . 196
Gastro-Intestinal Disturbances, Hydrozone and Glycozone in . . . 265
Gaylord, Harvey R., Investigation of Cancer . . . . . . . 549
Genito-Urinary Diseases, Mistaken Impressions Regarding . 563
Goler, George W., Rabies Epidemic in Rochester . . . 734
Gonococcus, Generalised Infections by . . . 180
Longevity of . . . . 315
Gonorrhea, Argonin in 408
Intra-urethral Injection . 196
Gould, George M., and Philadelphia Medical Journal . . . . . . 527
Gout, Pathogeny of . . . 186
Gram, F. C., Recent Epidemic of Measles in Buffalo . . . 39
Gunpowder, Removal of from Skin . 266

HANLEY, L. G., Hematoma of Broad Ligament . . 524
Hystero-Myomectomy . 833
Hanley, L. G., Melancholia Consequent upon Pelvic Disease . 832
Mental Aberration in Pelvic Disease 672
Pregnancy, Extra-Uterine . . . . . . 581
Removal of Pedunculated Fibroid . . 412
Health in Cuba . . . . . . . . . 60
Officer, Country . . . 488
Heart Disease, Suprarenal Capsule in, 526
Tobacco . . . . 194
Hemiplegia, Differential Diagnosis of . . . . . 200
Hemorrhoidal Operations, Local Anesthesia in . . . . . . . 688
Hemorrhoids, Excision of . . 651
For . . . . . . 919
Heroin, Incompatibilities of . . . . 443
Herpes, Caution Regarding . 523
Hopkins, Henry R., Practical Hygiene . 77
Howe, William A., Dermatitis Exfoliativa 481
Hurd, Arthur W., Paresis and Cerebral Syphilis . 629
Hydrogen Peroxide Bottles, Safety Valve Stopper for . . . . . 917
Hygiene, Practical . . . . 77
Report of Committee, Medical Society State of New York . . . . . . . . . 582

**Illustrations:**

Abbott, Frank W. Plate 763
Bainbridge: Fracture and Dislocation of Inferior Maxilla. Fig. 1, Fractures and Separation of Symphysis . 167
Fig. 2, Dressing in Situ . 168
Fig. 3, Irregularity of Teeth . . . . . . 169
Fig. 4, Second Right Incisor . . 169
Fig. 5, As Patient Appears Today . . . 170
Bissell: Formaldehyde Gas.
Fig. 1, Autoclave . . 497
Fig. 2, Regenerator . . 498
Fig. 3, Kuhn's Lamp . . 499
Fig. 4, Schering's Lamp . 500
Fig. 5, Schering's Disinfector . . . . . . . . 500
Fig. 6, Autoclave and Vaporiser . . . . . 501
Fig. 7, Paraformaldehyde Candle . . . . . . . 502

PAGE.

Cheesman: Cirsoid Aneurism. Fig. After Operation . . 398
College and Hospital Notes: Fig., Pan-Am. Emergency Hospital . . . . . 702
Dorr, Samuel G. Plate . . . 773
Editorial: Fig., Diploma Machine . . . 692
Howe: Dermatitis Exfoliativa.
  Fig. 1, Exterior . . . . 484
  Fig. 2, Interior . . . 485
Le Breton: Anesthesia. Fig. 1, The Hewett Inhaler . 88
  Fig. 2, The Bennett Inhaler . . . . 89
  Fig. 3, The Goldan Inhaler . . . . . 90
Lyon-Wright: Autochthonous Malaria. Plate, Map of Erie County . . . 249
Pan-American Exposition: Fig., Electricity Building 855
Potter: Pan-American Exposition. Fig. 1, Director-General . . . . 434
  Fig 2, Official Emblem 435
  Fig. 3, Service Building 436
  Fig. 4, United States Building 436
  Fig. 5, New York State Building . .. 437
  Fig. 6, Machinery Building 437
  Fig. 7, Tropical Court . 438
  Fig. 8, Manufacturers and Liberal Arts Building 438
  Fig. 9, Electricity Building . . . . . 439
  Fig. 10, Electric Tower . 439
  Fig. 11, Albright Art Gallery . . . 440
  Fig. 12, Temple of Music, 440
  Fig. 13, Stadium 441
  Fig. 14, Corner of Stadium . . . . . . . 441
  Fig. 15, Propylea . . 442
Reed, Charles A. L. Plate . 863
Smith, A. Everitt, Lupus Vulgaris. Fig. 1, Before Treatment 382
  Fig. 2, After Twelve Exposures 383
Stevens: Visual Plane, etc. Figs. 1, 2 and 3, Types of Crania 577
  Figs. 4, 5 and 6, Visual Planes . 578
  Figs. 7, 8 and 9, Types of Crania . . . . 579

PAGE.

Ullman: Malaria. Fig. 1, Hepatic and Splenic Enlargement . . . 894
  Fig 2, Enlargement of the Spleen . . . . . . 896
Wende, E.: Danger in Long Tube Nursing Bottles. Fig. 1, Irregular Porous Condition . . . 105
  Fig. 2, Porosity and Imperfect Seam . 105
  Fig. 3, Joint and Air Cavities . . . . . 106
  Fig. 4, Scraggy Elevations and Depressions . . 106
  Fig. 5, Elevations, Depressions and Imperfect Joint . . . . . 107
  Fig 6, Air Cells and Pocket, Inner Surface . 107
  Fig. 7, Pocket and Air Cell . . . . . . . 108

INFANT MORTALITY, Prevention of . . . . . . . . 120
Influenza in Buffalo . . . . . . . 528
Injuries of the Extremities, Conservtive Treatment of . . . . . . 793
Insane, Width of Pupils in . . . . 311
Intestinal Diseases of Children . 915
Introductory Lecture, U. of B. . 229
Iritis Glaucomatous . . . . . . . . 113

**Items:**

American Medical Association, Special Train for . . 872
Antikamnia Calendar . . . . 468
Bissell's Post-Graduate Course in Bacteriology 548
Breitenbach Co.'s Year Book . 548
Columbia Calendar . . . 468
Dr. W. E. Robbins's Library 380
Duffy's Malt Whiskey 792
Farbenfabriken of Elberfeld Company . 872
Glyco-Heroin Pharmacological Investigation . . . . . . . 548
M. J. Breitenbach Co.'s Loss by Fire 380
Maltine Company's Visiting List, 628
Medical Excursion, Paris and London . . . . . . . 944
Mellier Drug Company's Thermometer . . . 628
Scott & Bowne's Calendar 458
Tarrant & Co.'s Loss by Fire . 380
Unique Card-index System . . 468

PAGE.
Wheeler's Tissue Phosphates . 548
William R. Warner & Company, 792
Wyeth's Prepared Food . . . . 792

JACK, G. N., Grosser Animal Nature in Medicine 664, 743
Pathology of Asthma 815
Jacobson, Nathan, Sarcoma of the Tonsil . . . . . . 400
Johnson, J. Roberts, Autoinfection in Diseases of Children . . . . 305

KENERSON, VERTNER, Surgical Complications of Typhoid Fever . . . . . . 638
Kidney, Physiology of . . 898
King, Clarence, Functional Disorders of Digestion . . . . . . . 653

LAKE, A. D., Immunity in Tuber culosis . . . . . . . . . . 238

**Leading Articles:**

Advertising Crime . . . . . . 213
"A Few Kind Sentiments" . . 279
Antivivisectionists, For the . 54
Baalam's Ass Spake . . . . . 600
Buffalo as an Ism Center . . 53
Cancer, New Purpose of . . . 57
Collection of Garbage Again . 131
Commencement Day, U. of B. . 764
Dr. Reed's Presidential Address, Excerpts from . 922
Drug Standardisation Again . . 448
Ethical Schism in New York . . 125
Garbage, Collection of 56
Great Light from Philadelphia, A . . 52
How Shall Reciprocity be Established? . . 123
Medical Legislation at Albany 691
Medical Service Scandal in South Africa . . . . 55
Medical Society State of New York . . . . . 598
"Medicine as a Business" . . . 355
Modern Treatment of Epilepsy . 447
Mother Eddy's Coming To . . 604
Nature Renders a Verdict . . . 210
Newspapers and Medicine 50
New York Medical Journal Changes Publishers . 130
New York State Nurses' Association . . . . 694
Noisome Stench, A . . . . . 208
Philadelphia Medical Journal and New York . . . . . . 128

PAGE.
Physicians as Speakers . . . . 204
Professional Fees . . . . 353
Public Spirit and Politics . . . 212
Reciprocity and Medical Licenses Again . . . . . . . . . . 848
Small Pox and Vaccination . . 525
Society for the Prevention of Tuberculosis . . . . 769
Solomonesque Command, A . . 211
Some Newspaper Surgery . . . 354
Summer Drinks . . . . . . 767
Summer Noises . . . . . . 768
The Exposition . . . . . 847
Tom Johnson of Cleveland . . 850
Twentieth Century . . . 445
Two Meetings at St. Paul . . 921
University Day . . . 602
William Pepper Memorial Volume . . . . . . . . . . . . 278

Le Breton, Prescott, Anesthesia by Nitrous Oxide Gas and Ether 87
Legislature, Bills Before . . . 606
Life Insurance, Medical Examiner for . . . . . . . . . . . . . . . 392

**Literary Notes:**

American Medicine . . . . . . 626
Battle & Co's Charts . . . . . 944
Battle & Co.'s "Tortures of the Gout" . . 76
Battle's Anatomical Drawings . . . . . . . . 304, 467, 712
Buffalo Academy of Medicine Prize . . . 547
Bulletin of the Medical Sciences, 712
Challen's List of Periodicals, etc. . . . . . . . . . . . . 74
Electrical Review . . . . . . 547
Essentials of Hematology . . 74
Ewing on the Blood . . . . . 712
Facts and Figures, New York Pharmacal Association . . . 943
Fox's Atlas of Skin Diseases . . 943
Grosvenor Public Library . 872
Guide to Carlsbad, Dr. Arany's . 943
International Directory of Laryngologists and Otologists . . 73
International Journal of Surgery Company's New Publications, 228
Koenig's Text-Book of Special Surgery . . . . . . . . . 547
Listerine . . . . . . . . . . . 152
Medical Bulletin, University of Pennsylvania . . 712
Medical Mirror Announcements . . . . 228, 872
Medical Mirror Prize Essays on Tuberculosis . . . . . . . . . 75

PAGE.

Mosquitoes and Malaria . . 75
N. Y. State Reformatory Year-Book . . . . . . . . . . . . 75
New York Post-Graduate Medical School Announcement 304
New York State Journal of Medicine . . . . . . 547
New Zealand Medical Journal . 304
Northwestern Lancet . . . . 547
Polk's Medical Register . . 872
Palisade Manufacturing Company's Papyrus—Ebers . . 791
Pan-American Exposition Booklet . . . . . . 626
Prize Essay on Dangers from Quackery . . . . . . . . . 626
Quarterly Journal of Inebriety . 944
Recipes for Infant Food . . . 152
Reed & Carnrick's Brochure on Blood . . . . . 379
Reed & Carnrick's Laboratories, 76
Reed's Text-Book of Gynecology . . . . . . . . . . . 467
Standard Medical Directory . . 790
Stylus and Interstate Medical Journal . . . . . . 625
Texas Medical Gazette . 625
Therapeutic Gazette, Editor's Removal . . 944
Therapeutics of Pepto-Mangan (Gude) . . . . . 467
W. B. Saunders & Company's New Publications . . . 227
Warner's Therapeutic Reference Book . . 74, 304
Western Reserve University's News-letter . . . . . . . . . 468

London, Housing of Poor in . . . 606
Long, B. G., Prevention of Tuberculosis . . . . . . . . 880
Lupus Vulgaris Treated by X-ray . 381
Lyon-Wright, Autochthonous Malaria in Buffalo . . . . . . . 245

MALARIA, Autochthonous, in Buffalo . . . 245
Involvement of Nervous System in . . 344
Three Varieties Observed in Buffalo . 892
Maloney, Frank W., Examiner for Life Insurance . . 392
Maltine Company Refuses to Supply Department Stores . . 607
Mann, Matthew D., Puerperal Infection . . . . . . . . . . . . . . 1

PAGE.

Mann, Matthew D., Removal of Bladder for Malignant Disease . . . 900
Matzinger, H. G., Blood Examinations . . . . 319
Measles, Recent Epidemic of, in Buffalo . . . . . 39
Medical Examiners, Appointment of, 705
Glimpses of Lands Across the Seas . . . 255
Meyer, P. H., Spectacles and Eye-glasses from the Optician's Standpoint . . . . . . . . . . . . 803

**Miscellany**:

Army Medical Boards . . . . 627
J. B. Lippincott Company's New Site . 227
Maltine Company's Family Tree, 227
New York Pharmacal Association and the Census . 627
Rudolph Virchow Fund . . . 791
St. Paul Meeting and Yellowstone Park . . . . . . 791
William R. Warner & Company's World's Fair Award . . . 227

Myalgia . . . . . . . . . . . . . 276
Mynter, Herman, Prostatic Hypertrophy . . . . . . . . . . . . 16

NEURALGIA, Trigeminal . . 276
New York Academy of Medicine, Library Report . 527
New York State Medical Association, Reason for Existence 925
Tribune and Professional Standards . . . . . 696
Nipples, For Fissured . . . . 275
Nose, Fractures of . . 811
Nursing Bottles, Danger in Long Tube . . . . . . . . . . . 104

OBSTETRICS from a Gynecological Standpoint . . . 260

**Obituary**:

Abbott, Frank W. . . . . 775
Armstrong, J. Stone . . . 699
Ashurst, John, Jr . . . . . . 64
Banta, Westervelt . . . . . 930
Brown, Ulysses Higgins . 531
Browning, William Webb . . . 359
Burritt, Franklin . . . . . 217
Chapman, James . . . 699
Christopher, Hiram . . . . . 610
Clippinger, John W . . . . 699

| | Page. |
|---|---|
| Cole, Richard Beverley | 531 |
| Curtiss, Romaine J | 452 |
| Cutler, J. L | 284 |
| DaCosta, J. M | 217 |
| Daly, William H | 929 |
| Dorr, Samuel G. | 772 |
| Draper, William H | 775 |
| Dunglison. Richard J | 698 |
| Fruitnight, J. Henry | 531 |
| Groess, Edward L. | 930 |
| Haggard, William D. | 699 |
| Hanks, Horace Tracy | 360 |
| Herrick, Henry J | 609 |
| Hill, Henry Clay | 610 |
| Ingals, Ephraim | 452 |
| Jarvis, George C | 859 |
| Lewis, George W. | 63 |
| McGuire, Hunter | 217 |
| Miller, John H | 452 |
| Montfort, Robert V. K. | 531 |
| Nichell, Henry | 609 |
| Noyes, Henry Drury | 360 |
| Parkhurst, Wm. H | 531 |
| Perkins, Maurice B. | 929 |
| Phillips, Corydon J | 610 |
| Pleuthner, Wm. H | 531 |
| Purdy, Charles Wesley | 531 |
| Purple, Samuel Smith | 284 |
| Robinson, John W | 284 |
| Roenmelt, Charles | 284 |
| Sayre, Lewis A | 217 |
| Scott, Preston B | 217 |
| Skene, Alexander J. C. | 62 |
| Sibley, Wallace A | 360 |
| Smith, Alonzo T | 699 |
| Squibb, Edward Robinson | 359 |
| St. John, Charles A. | 930 |
| Stillé, Alfred | 217 |
| Streeter, Buel Goodsell | 134 |
| Taft, Charles Sabin | 452 |
| Turnbull, Laurence | 360 |
| Warner, William R. | 775 |
| Welles, Samuel Russell | 63 |
| Wheeler, Thomas Brown | 530 |

Odell, Governor, and Special Bills . . 852
O'Gorman, Francis M., Antitoxin in Cerebro-Spinal Meningitis . . . . 177
Opium Habit in the East . . 122

PAN-AMERICAN EXPOSItion . . . 434
Notes 854, 926
Pan-Am. "Is" in Buffalo . . 854
Paresis and Cerebral Syphilis . . . 629
Park, Roswell, The Nature of the Cancerous Process . . . . . . 873
Pelves, Injury to Skull in Narrow . . 118
Peritoneum, Toilet of . . . . . . 405

**Personal :**

Almy, Leonard B.; Phelps, A. M.; Grove, Robert K . . . . . . 60
Angell, Edward B.; Park, Roswell; Cary, Charles; Stone, William Ridgley; Smith, Chauncey Pelton; Ullman, Julius; Downey, Martin J.; Dunham, S. A.; Lewis, Daniel, 451
Angell, Edward B.; Curran, Richard; Blumer, George; Potter, William Warren . . . . . 771
Brown, Ira S.; Pitkin, John T.; Potter, William Warren; Carstens, J. Henry; Vaughan, Victor C.; Jelks, James T. . 450
Dunham, S. A.; Love, F. W.; Williams, H. T. . . . . . . 134
Forsyth, E. A.; Blaauw, E. E.; Kenerson, Vertner; Wasdin, Eugene; Kuhlman, Helene J. C.; Russell, Nelson G.; Brown, Ira C.; King, James E.; Bennett, A. G.; Dowd, J. Henry 698
Fronczak, Francis E . . 132
Granger, R. B.; Vander Veer, A.; MacDonald, Arthur; Beck, W. F.; Benoit, Marie Louise; Hill, Henry W.; Fisher, M. E. . 61
Griffith, J. D.; Christian, Frank L; Millener, F. H.; Beck, W. F; Elsner, Henry L.; Suiter, A. Walter; Stoddard, Thomas, 928
Guiteras, John; Goldberg, S.; Mead, Harry; Le Breton, Prescott . 217
Howe, Lucien; McFarland, Joseph; Burdick, James T.; Van Peyma, P. W.; Renner, W. Scott . . . . . . . . . . 608
Jacobi, Abraham . 770
Kuhlman, Helene J. C.; Brewer, Geo. E.; Macbeth, Albert H.; Searls, M. B.; Hains, F. G.; Clausius, M. F.; Jack, G. N.; Smith, Eugene A. . 133
Miller, John A.; Cameron, J. W.; Crance, Charles T.; Dunham, S. A.; Van Peyma, P. W.; Grant, John H.; Ruggles, E. Wood; Spiller, W. G. . . 283
Pedersen, Victor C.; Lyon, Irving P.; Renner, W. Scott; Rolph, R. T . . . 62
Peterson, Frederick; Brady, William A.; Peck, Geo. S.; Knopf, S. A.; Foster, H. A.; Ullman, Julius; Woehnert, A. E.; Loveland, B. C.; Lewi, M. J. . . . . 859

PAGE.
Reed, Charles A. L.; Stevens, George T.; Johnson, J. C. . . 529
Renner, W. Scott . . . . . 857
Riester, Julian A.; Suiter, A. Walter; O'Neill, J. M.; Deane, W. J.; Smiley, J. L.; Stanton, William; Benedict, A. L. . . 359
Scudder, Charles L.; Grant, John H.; Himmelsbach, G. A.; O'Gorman, Francis M.; Patchin, R. A. . . . . . . 772
Shurly, E. L.; Foster, H. A.; Howe, Lucien; Downey, Martin J.; Heath, W. H.; Wilson, Nelson W.; May, Wm. B. 358
Smith, Eugene A.; Lewis, Daniel, 696
Tolfree, Herbert M.; Ricketts, Edwin; Waterman, L. G. . . 929
Wasdin, Eugene; Barrows, F. W.; Haggard, Wm. D., Jr.; Russell, N. G.; Lothrop, Thomas; Aaron, Charles D.; Mead, Captain Harry; Carlisle, Chester L. . . . . . . . 530
Waterman, J. Hilton; Mann, Matthew D.; Wilcox, DeWitt G.; Noble, Harriet Watson; Griswold, V. M.; Ullman, Julius; Shaw, O. C. . . . . . . 609
Wende, Ernest; Hubbell, A. A.; Lothrop, Thomas; Pohlman, A. G.; May, Wm. B.; McMurtry, L. S. . . . . . . . . . 282
Wende, Ernest; Love, I. N.; Winne, Charles K.; Mann, Matthew D.; Krauss, William C. . . . . . . . . . . . 216
Wilson, Nelson W.; Winne, Charles K.; Mann, Matthew D.; Farr, Charles W.; Ernest, J. Glen; O'Donnell, W. J.; Gould, J. P. G.; Wende, Ernest; Gipple, B. A. . . . . . 697
Wiltse, Ralph H.; Taggart, Jas. A.; Pryor, John H.; Burrows, Lorenzo, Jr.; Shriver, Eli, Jr.; Durand, C. F.; Pohlman, Julius; Miller, John A.; Griffin, William T.; McAdam, L. J. . . . . . . . 858
Woodruff, Timothy L.; Grant, John H . . . . . . . . . 215

Pharmacy Law, New State . . . 528
Phthisis, Irritating Cough of . . . . 195
Physician Fined for Contempt . . . 607
The . . . . . . . . . . 171
Plague and Rats . . . . . . . 58
Pneumonia, Creosotal in . . . . . 192
Delayed Resolution in . 273

PAGE.
Potter, William Warren, Pan-American Exposition . . . 434
Posterior Columns, Non tabetic Lesions of . . . . . . . . 197, 203
Pregnancy in Fimbriated Extremity . 119
Signs of . . . . . 118
Toxemia of . . . . . . 736

**Progress in Medical Science:**

Genito-Urinary and Syphilitic Diseases, by Byron H. Daggett . . . . 113, 188
Neurology, by William C. Krauss . . . . 197
Obstetrics and Gynecology, by William Warren Potter . . . 116
Ophthalmology, by Alvin A. Hubbell . . . . . 109
Therapeutic Notes, by Nelson W. Wilson . . . . 192, 273, 918

Prostatic Hypertrophy, Treatment of . . . . . . . . 16, 274
Protargol, Manner of Using . . . 597
Proteids, Analysis of, in Stomach Contents . . . . . . . . . . 669
Puerperal Infection . . . . . . 1, 117

QUININE, Abuse of . . . . . 98

RABIES . 724
Epidemic of, in Rochester, 734
Epidemic of . 215
Epidemic of, in Buffalo, Recent . . . . 713
Rasbach, F. B., Skin Grafting . . . 799
Ravenal, Rabies . . . 724
Reed & Carnrick's Clinical Laboratory . . . . . . . . . . . . . 606

**Reviews:**

Abrams: Diseases of the Heart, 942
Anders: Practice of Medicine . 622
Ayers: Physical Diagnosis in Obstetrics. . . . . 787
Bacon: Manual of Otology 544
Ballenger: Diseases of Eye, Ear, Nose and Throat . . . . . 620
Baudry: Injuries to the Eye 299
Beck: Fractures . . . . . . 288
Berkley: Mental Diseases . 460
Blakiston: Physician's Visiting List. 465
Böhm-Davidoff: Text-Book of Histology . . . . . . . . . 617

PAGE.
Brown: Diseases of the Nose and Throat . 143
Buck: Reference Handbook, Vol. I, 461; Vol. II. . . . . 941
Cabot: Physical Diagnosis. . . 618
Cattell: International Clinics, Tenth Series, Vol. II., 295; Vol. III., 545; Vol. IV., 869; Eleventh Series, Vol. I. 869
Chapman: Heart Disease in Childhood 465
Cheyne-Burghard: Manual of Surgical Treatment, Vol. III., 223; Vol. IV. . . . . . 935
Christison: Brain in Relation to Mind . . . 373
Coplin: Manual of Pathology . 221
Cotton: Anatomy, etc., of Infancy and Childhood 377
Crile: Surgery of Respiratory System . . . . 787
Culbreth: Materia Medica and Pharmacology . . . 619
Cullen: Cancer of the Uterus 867
Cushny: Pharmacology and Therapeutics . . 709
DaCosta: Modern Surgery. . . 620
Davis: Refraction of the Eye . 69
Obstetric and Gynecologic Nursing . . 940
Deaver: Surgical Anatomy, Vols. I. and II. . . . . . 457
Treatise on Appendicitis . 287
Dorland: Medical Dictionary. 368
Duane: Dictionary of Medicine . . . . 299
Dunglison: Dictionary of Medical Science. . . 615
Dunham: Normal Histology . 148
Durck: Atlas of Pathologic Histology . . . 71
Egbert: Hygiene and Sanitation . 622
Ellis: Psychology of Sex. . . 540
Einhorn: Diseases of the Intestines. . . . . . 543
Evans: Text-Book of Obstetrics. . . . 710
Fischer: Infant Feeding. . . 782
Fowler: Treatise on Appendicitis. 938
French: Food for the Sick. 300
Friedrich-Curtis: Rhinology, Laryngology and Otology . 537
Gardiner: Care of the Consumptive . . . 707
Garrigues: Diseases of Women, 616
Golcbiewski: Atlas of Accident Diseases . . . . . . . 296

PAGE.
Gould: Student's Medical Dictionary. . . . . . . 464
Gould: Suggestions to Medical Writers . . . . . . . . 291
Gould: Year Book for 1901 . 621
Grandin-Jarman: Practical Obstetrics. . . . 869
Green: Pathology and Morbid Anatomy. . . . 374
Hare: Practical Therapeutics. . 378
Progressive Medicine, Vol. III., 1900, 298; Vol. IV., 617; Vol. I., 1901. 939
System of Practical Therapeutics, Vols. I and II, 706; Vol. III. . . . . . 782
Hartridge: Refraction of the Eye . . . . . . . . . 146
Hemmeter: Diseases of the Stomach . . . 67
Hopkins: Treatise on Fractures, 624
Howell: Pocket Medical Formulary . . . . . . 789
Howell: Text-Book of Physiology, Vol. I., 622; Vol. II. . . 708
Hutchison: Food and Dietetics, 785
Hyde: Syphilis and Venereal Diseases . . . . . . . . 293
Ingals: Diseases of the Chest, Throat, etc. . . . . . . . . 378
Jackson: Diseases of the Skin . 539
Jacobi: Festschrift . . 542
Keyes-Chetwood: Venereal Diseases . . . . . . . . . . . 145
King: Manual of Obstetrics . . 300
Kleen: Diabetes Mellitus . 147
Kyle: Diseases of Nose and Throat. . . . . . . . . 941
Lea: Medical News Visiting List. . . . . . . 377
Leroy: Essentials of Histology, 544
Levy-Klemperer: Clinical Bacteriology . . . . . . . 146
Lewis: Obstetric Clinic . . . 708
Lincoln: Sanity of Mind. . 783
Lloyd: Stringtown on the Pike, 464
Loeb: Comparative Physiology of Brain . . . . . . . . . . 789
Lutaud: Manual of Gynecology, 151
Marsh-White: Records of Mutual Life Insurance Co. . . . 541
Martin: Chemistry and Physics, 295
May: Diseases of the Eye 374
Moore: Laboratory Directions in Bacteriology . . . . . . . 543
Monell: Medical Electricity 788
Musser: Medical Diagnosis . . 459
Mynter: Appendicitis . . . . 619
Nettleship-Campbell: Diseases of the Eye . . . . . . . . . 148

PAGE.
O'Brien: Medical and Surgical Nursing . . . 463
Ogden: Examination of the Urine 372
Oppenheim: Diseases of the Nervous System 868
Parke, Davis & Co.: Manual of Therapeutics . . . . . . 538
Pyle: Personal Hygiene. . 376
Reed: Text-Book of Gynecology, 862
Report of Commissioner of Education, 1897–1898, 224; 1898–1899 . . . 623
Presbyterian Hospital, Vol. IV. . . . . . 225
State Board of Health of New York, 1898 623
Roger: Introduction to Study of Medicine 784
Sajous: Cyclopedia of Practical Medicine, Vol. V. . . . . . . 70
Salinger: Modern Medicine 535
Schaefer: Atlas of Gynecology . 296
Scudder: Treatment of Fractures . . . . . . . . 288
Senn: Pathology and Treatment of Tumors. 865
Shoemaker: Materia Medica and Therapeutics. . . . . 709
Shurly: Diseases of the Nose and Throat. . . 364
Simon: Clinical Diagnosis . . . 220
Snell: Eye Accidents . . . 296
Solis-Cohen and Eshner: Essentials of Diagnosis . . . 150
Stedman: Twentieth Century Practice, Vol. XX. 462
Stengel: Text-Book of Pathology . . . . . . 621
Stephens: Perfect Eyes . . 373
Stimson: Operative Surgery. 369
Stimson: Fractures and Dislocations. . . 542
Stoney: Bacteriology, etc., for Nurses. 465
Sturgis: Manual of Veneral Diseases. . 937
Sexual Debility in Man. 613
Taylor: Diseases of Children, 788
Taylor: Genito-Urinary and Venereal Diseases . . . 370
Taylor: Sexual Disorders . . . 141
Thomas: Sickroom Dietary . . 465
Thompson: Text-Book of Practical Medicine 375
Tiard: Diseases and Symptoms. 463
Transactions:
American Association of Obstetricians and Gynecologists, Vol. XII. . . 142

PAGE.
American Laryngological Association . . . . . . . 545
American Otological Society . . . . . . . . 870
American Surgical Association, Vol. XVIII. . . . . 544
Medical Association of Central New York, Vol. VI. . 225
Medical Society of the State of New York, 1900 . 540
Southern Surgical and Gynecological Association, Vol XII. . . . . . . . . . 224
Texas State Medical Association, 1900 . . . . 624
Treat: International Medical Annual, 1900, 68; 1901 . 786
Valentine: Treatment of Gonorrhea . . . . . . . . . 290
Warren: Surgical Pathology and Therapeutics . . 149
Wainwright: Urinary Diagnosis and Treatment . . . . . . 780
Whitehead: Anatomy of the Brain . . . 150
Williams: Diseases of Infancy and Children . 371
Uterine Tumors, Pathology and Treatment . . . . . 939
Wood: Therapeutics . . 616
Woody: Medical Chemistry . 300

Rheumatism, Gonorrheal . . . . . 92
Rheumatic Manifestations, Treatment of . . . . . . . . 477
Rhinitis, Hypertrophic, Treated with Adrenalin . . . . . . . . . . 833
Ringworm, for . . . . . . . . . 274
Richy, H. A., Treatment of Rheumatic Manifestations . . . . . 477
Roseola, Typhoid Bacilli from . . . 684
Ruggles, Longevity of the Gonococcus . . . . . . . . . . . . . . 315

SARCOMA of the Tonsil . . . . 400
Senility and Senile Dementia, Medico-Legal Aspects of . 558
School Buildings, Ventilation of . . 695
Skin Grafting . . . 799
Smith, A. Everitt, Lupus Vulgaris Cured by X-ray. . . . 381
Removal of Burnt Powder from the Skin. . . . . 266
Smith, Chauncey Pelton, Injuries of the Extremities. . . 793
Smith, Eugene A., Cholelithiasis from Surgical Viewpoint 385
Early and Late Operations for Appendicitis . . 23

PAGE.
Smith, Eugene A., Introductory Lecture, U. of B . . . . . . . . . 229

**Society Meetings:**

American Academy of Medicine, 777
American Association of Obstetricians and Gynecologists . . . . . . . . . . . . 64, 218, 933
American Congress of Tuberculosis . . . . 610, 932
American Dermatological Association . . . . . . . 861
American Electro - Therapeutic Association. . 139
American Gynecological Society, 933
American Laryngological, Rhinological and Otological Society, 932
American Microscopical Society, 138
American Medical Association 777
American Medical Editors' Association . 860
American Medico-Psychological Association . . . 860
American Pediatric Society . 777
American Proctological Society 777
American Public Health Association. . . . . 219, 360, 933
American Therapeutic Society . 140
Association of Confederate Medical Officers 701
Buffalo Academy of Medicine, . . . 66, 134, 219, 285, 361, 453, 532, 611, 700, 776, 860, 931
Canandaigua Society of Physicians . . 611
Central New York Homeopathic Medical Society . 699
Erie County Medical Association . . . . . . 454
Esculapian Club . . . 218, 285
International Congress of Nurses . . 700
Keuka Lake Medical Association . 138
Medical Association of Central New York . . . . 140, 218, 933
Medical Society of the County of Erie . . . . . . 453, 861, 932
Medical Society of the State of New York . . 360, 454, 778, 930
Medical Union . . . 284, 532
Mississippi Valley Medical Association . . . . . . 65, 285, 861
National Association for Study of Epilepsy . . . . . 860
National Confederation of State Medical Examining Boards 776
New York State Association of Railway Surgeons. . . . . 284

PAGE.
New York State Medical Association, Fourth District Branch . . . . . . . . . 932
Niagara Falls Academy of Medicine . 285, 361, 452, 533, 610, 778
Pan-American Medical Congress, . . . 453, 610
Physicians' Club . . 452
Rochester Academy of Medicine, . . . . . . . . . 139, 532
Röntgen Society . . . . 932
Southern Surgical and Gynecological Association 220, 362
St. Louis Academy of Medicine . . . . . . . . . . . 362
Tri-State Medical Society . . . 218
Tuberculosis Congress . . 931
Western Ophthalmologic and Oto-Laryngologic Association, . . . . . . . . . 612, 778

**Society Proceedings :**

Buffalo Academy of Medicine, . . . . . . 187, 750, 901
Section on Surgery . . . . . . . . . 187, 413, 595
Section on Ophthalmology, Otology, Rhinology and Laryngology . . . . 268, 907 347, 422, 515, 590, 680, 834
Section on Obstetrics and Gynecology, 272, 351, 429, 593, 755, 841, 914
Section on Pathology . 343, 520, 673, 750, 911
Section on Medicine . 344, 416, 511, 586, 676, 758, 838, 903
Clinical Society, Buffalo General Hospital . 433
Thirteenth International Medical Congress . . 180
Medical Association of Central New York . . . . . 335
Medical Society of the County of Erie 43, 506, 761, 844

Spectacles and Eyeglasses, Making and Fitting of 803
Spitting, Ordinance Against 695, 853
Stanton, William, The Physician 171
Stephenson, F.H., Senility and Senile Dementia 558
Stevens, George T., Pose of Body, Type of Cranium and Visual Plane, 576
Streets, Bad Condition of . . . . 695
Suprarenal Extract . . 689
Syphilis, Signs of Inherited . . . 113
Treatment of . . . . . . 687

PAGE.

TENDON REFLEXES, Nature of . . . . . . . . . . . 202
Tonic, Nerve . . . . . . . . 276
Trional Mixture . . . . 193
Trusses, Note on Fitting . 491
Tuberculosis, For Night Sweats in . 274
Immunity in . . . . . 238
Restriction of . . . 357
Prevention of . . . 880
Typhoid Fever, Surgical Complications of . . . . . . . . . . 638

ULLMAN, JULIUS, Malaria Representing Three Varieties Observed in Bufalo . 892
University of Pennsylvania, Curriculum of Medicine at . . . . . . 526
Urethral Chills, To Ward Off . . . 919
Urethritis, Mercurol in . . . . . . . . 263
Urticaria, Sodium Phosphate in . . . 194
Uterus, Early Recognition of Cancer of . . . . . . . . . . 352
Rupture of . . . . . . . . . 116

PAGE.

VEEDER, A. E., Note on Fitting Trusses . . . . 491
Visual Plane, Type of Cranium and Pose of Body . . . . . 576

WANDER DAYS, A Doctor's . 529
Wende, Ernest, Danger in Long-Tube Nursing Bottles . . . . 104
Wende, Ernest, Recent Epidemic of Rabies in Buffalo . . . . . 713
Whooping Cough, for . . . . . . . . 920
Wilson, Nelson W., Argonin in Gonorrhea . . . . . . . . . . . . . . 408

X-RAYS, Medico-Legal Relations of . . . . . . . . . . . . . 121

YOUNG, A. A , Formaldehyde . . 553

